# Supplementary material for: Kuwanon C Inhibits Tumor Cell Proliferation and Induces Apoptosis by Targeting Mitochondria and Endoplasmic Reticulum
Source: Int J Mol Sci. 2024 Jul 30;25(15):8293. doi: 10.3390/ijms25158293 (PMC11312418; doi:10.3390/ijms25158293)
Supplement: Supplementary file 1 [file ijms-25-08293-s001.zip › ijms-3075145-supplementary.pdf]

Supplementary Figures and Tables

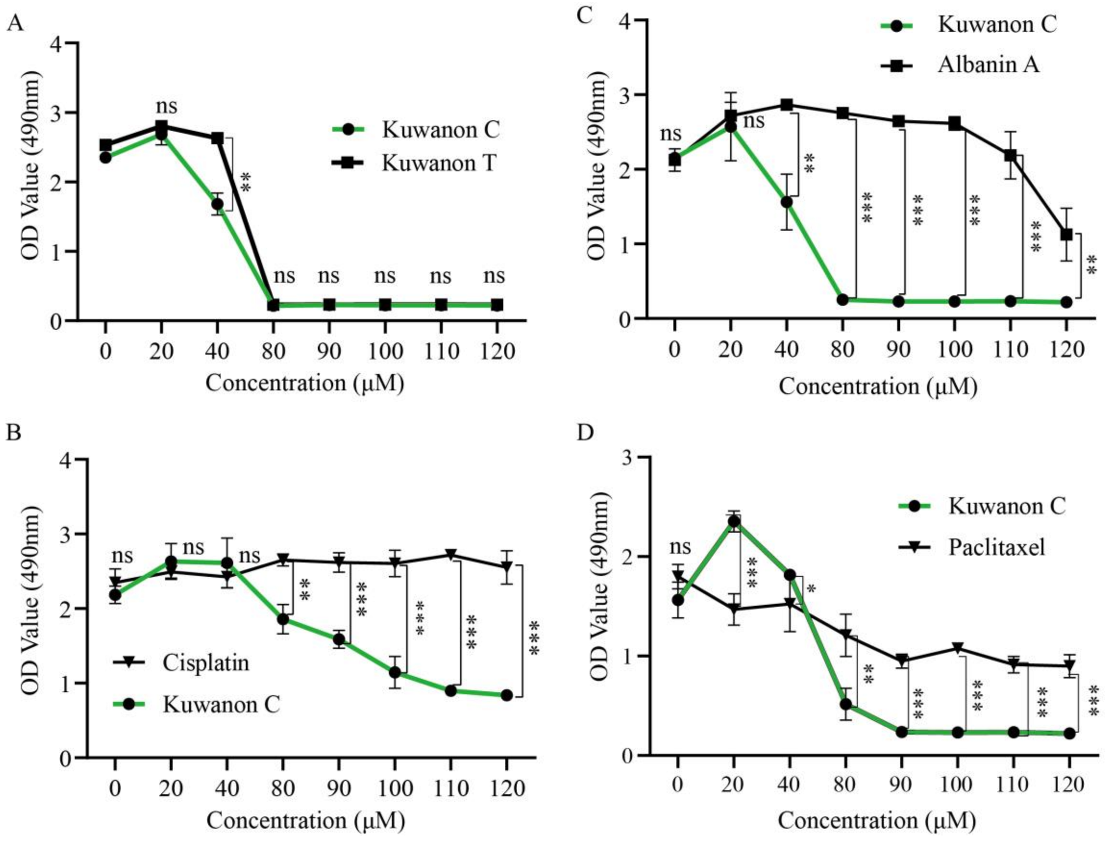

Figure S1. Comparison of Hela cell viability by Kuwanon C and Kuwanon T and antitumor drugs

(A) The impact of the isomeric form of Kuwanon C, Kuwanon T, on cell proliferation in Hela cells after 24 hours of treatment. (B, D) Comparison of the effect of Kuwanon C on the viability of Hela cells with clinically used antitumor drugs Cisplatin and Paclitaxel. (C) Comparative evaluation of the anti-tumor proliferative capacity between Kuwanon C and Albain A, which contains one isopentenyl group, in Hela cells treated for 24 hours. Each bar represents the mean ± SD of three independent experiments. Statistical significance is indicated by *P < 0.05, **P < 0.01, ***P < 0.001 and ns: not significant.

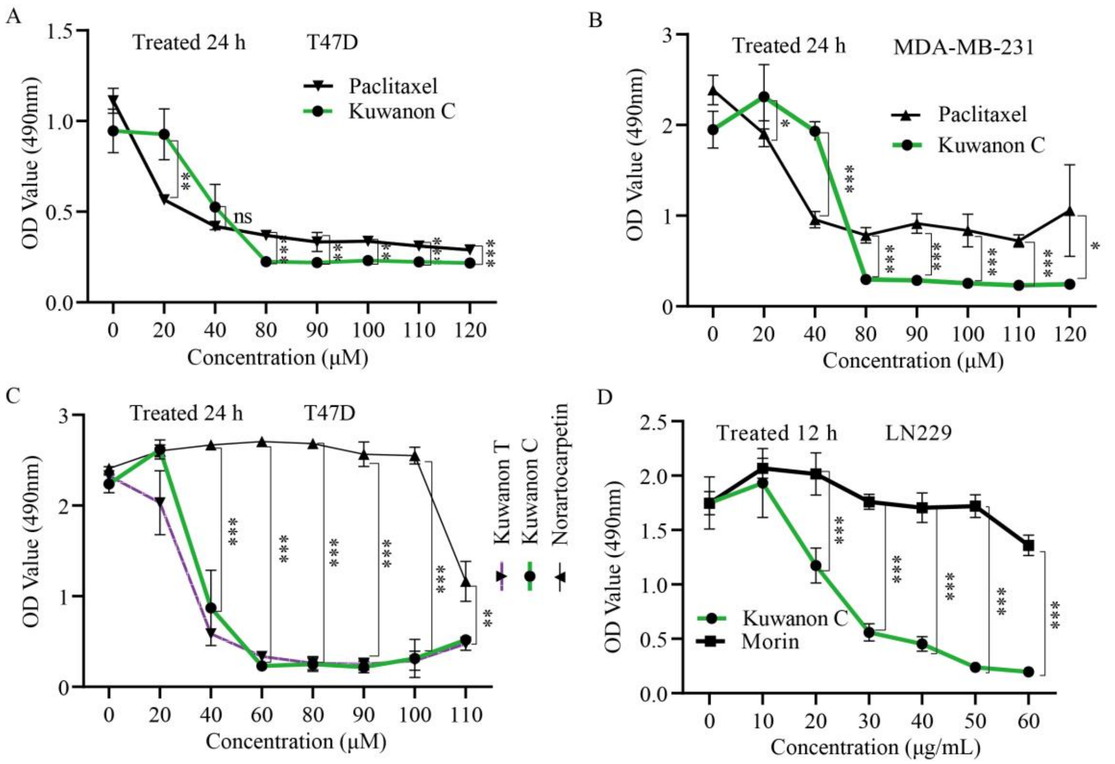

Figure S2. Suppression of viability by Kuwanon C in breast cancer cells and brain tumor cells

(A, B) Comparison of the effects of Kuwanon C and Paclitaxel on the viability of breast cancer cells T47D and MDA-MB-231 cells. (C) Differences in the viability of breast cancer cells depending on the number and position of isopentenyl groups were compared on T47D cells. (D) Effect of Kuwanon C on the viability of brain tumor cells LN229. Each bar represents the mean ± SD of three independent experiments. Statistical significance is indicated by *P < 0.05, **P < 0.01, ***P < 0.001 and ns: not significant.

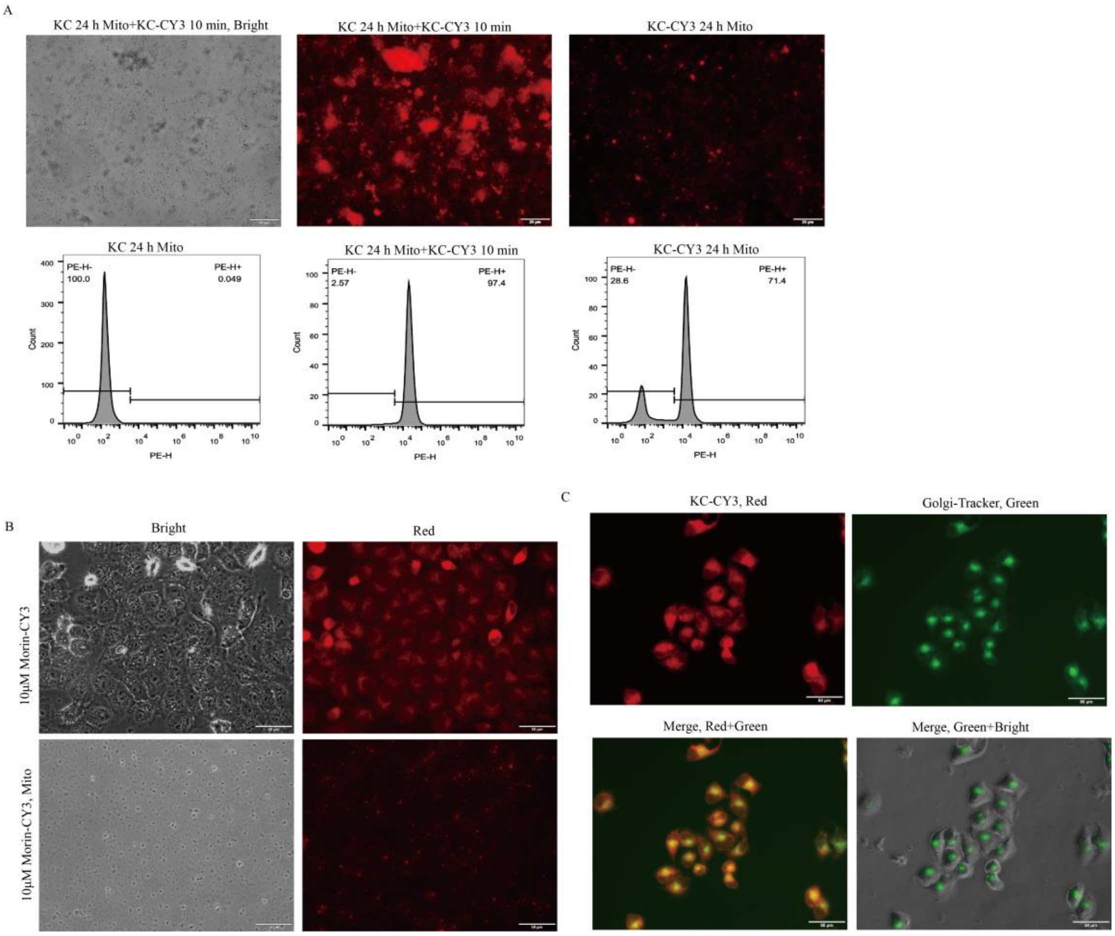

Figure S3. Binding target site analysis of Kuwanon C

(A) Cellular mitochondria extracted from Kuwanon C and Kuwanon C-CY3-treated Hela cells, respectively. The mitochondria extracted from the Kuwanon C-treated group were co-incubated with Kuwanon C-CY3 and microscopically observed to emit red fluorescence, and the fluorescence signal intensity of the mitochondria was detected to be enhanced by flow cytometry. (B) Fluorescence microscopy observed red fluorescence on mitochondria after treatment of Hela cells with CY3-labeled Morin. (C) Kuwanon C-CY3 overlapped with Golgi fluorescence-specific probes bound in Hela cells.

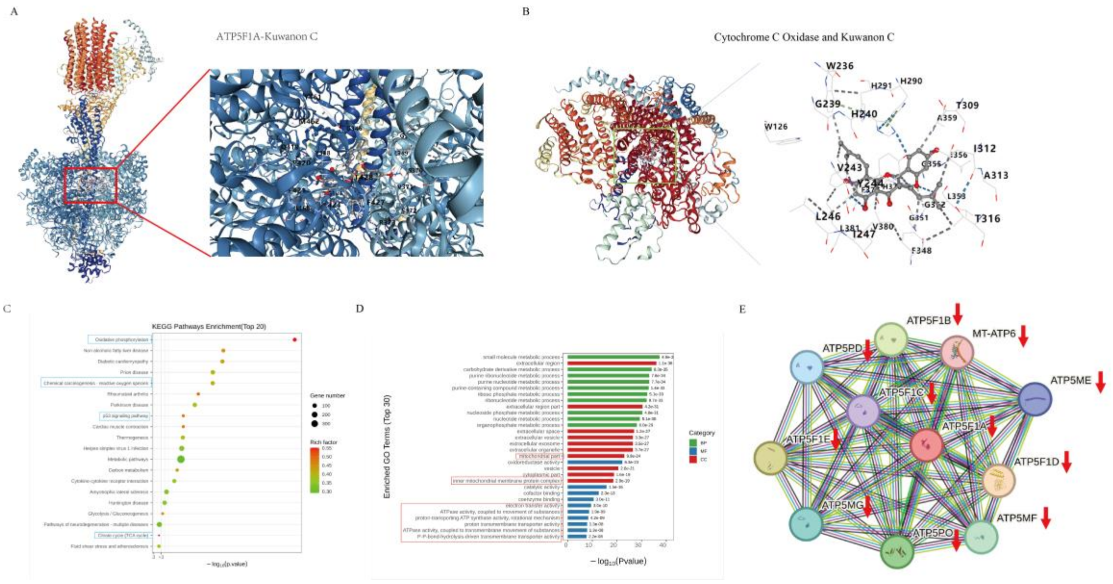

Figure S4. Impact of Kuwanon C on ATP production in Hela cells

(A, B) Molecular docking analyses using CB-DOCK2 were performed to examine the binding interactions between Kuwanon C (KC) and ATP5F1A as well as cytochrome oxidase. (C) KEGG pathway enrichment analysis of transcriptomic data after Kuwanon C treatment of Hela showed that oxidative phosphorylation, ROS, and TCA cycling were affected in the top twenty pathways. (D, E) Mass spectrometry and transcriptome analysis results demonstrated the potential binding of Kuwanon C to ATP synthase protein and cytochrome oxidase protein. and Transcriptional data were analyzed for expression levels of genes on pathways associated with ATP production. (Red downward arrows show reduced levels of gene expression).

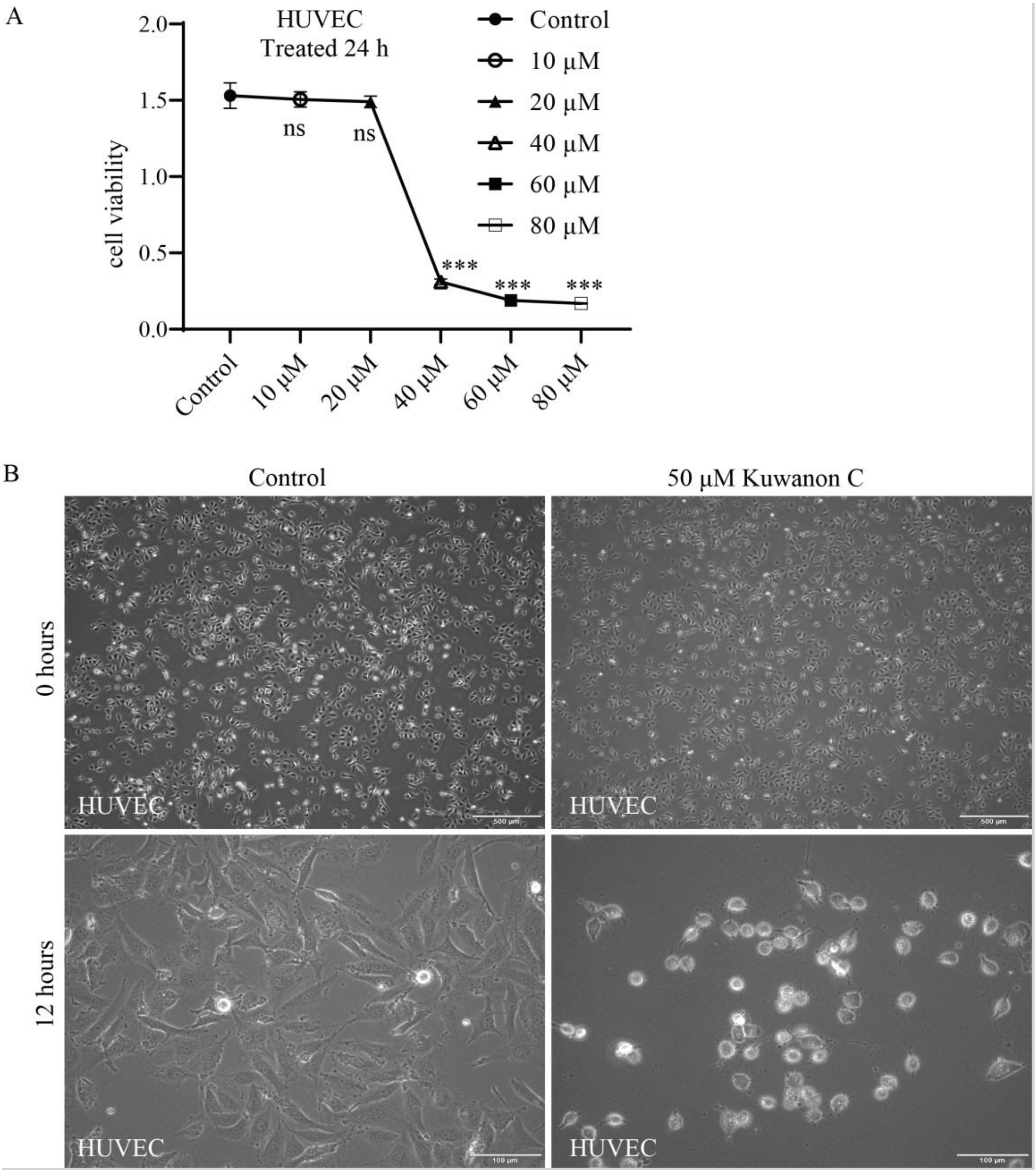

Figure S5. Effect of Kuwanon C on normal cells

(A) MTS detection of the effect of Kuwanon C on the viability of HUEC cells. (B) Microscopic observation of the effect of Kuwanon C on the morphology of HUVEC cells.

Supplemental Table S1. Molecular docking analysis of Kuwanon C and CDK1

CurPocket ID	Vina score	Cavity volume ($Å^3$)	Center (x, y, z)	Docking size (x, y, z)	Contact residues
**Kuwanon C and CDK1**	-10.1	1535	29, -66, 190	22, 22, 29	**Chain A**: ILE10 GLY11 GLU12 GLY13 TYR15 VAL18 ALA31 MET32 LYS33 VAL64 PHE80 PHE82 LEU83 SER84 MET85 ASP86 LYS88 LYS89 GLN132 ASN133 LEU135 ALA145 ASP146

Supplemental Table S2. Molecular docking analysis of Kuwanon C with ATP5F1A and cytochrome C oxidase

CurPocket ID	Vina score	Cavity volume ($Å^3$)	Center (x, y, z)	Docking size (x, y, z)	Contact residues
**ATP5F1A and Kuwanon C**	-9.8	2983	222, 184, 231	31, 22, 28	**Chain B**: LEU156 VAL367 GLY368 LEU369 SER370 VAL371 SER372 ARG373 LYS391 **Chain F**: VAL163 GLY164 VAL167 LEU168 GLY346 TYR348 GLN419 PRO420 PHE421 ALA424 PHE427 THR428 TYR461 MET462
**Cytochrome C Oxidase and Kuwanon C**	-10.4	17502	334, 330, 259	29, 35, 35	**Chain A**: TRP126 TRP236 GLY239 HIS240 VAL243 TYR244 LEU246 ILE247 HIS290 HIS291 THR309 ILE312 ALA313 THR316 PHE348 GLY351 GLY352 LEU353 GLY355 ILE356 ALA359 HIS376 PHE377 VAL380 LEU381
